# Rare diseases in Brazil: a nationwide analysis of the diagnostic odyssey

**DOI:** 10.1007/s12687-026-00939-y

**Published:** 2026-09-15

**Authors:** Bibiana Mello de Oliveira, Monique Sartori Broch, Carolina Peçaibes de Oliveira, Angélica Piovesana, Claudia Fernandes Lorea, Mariana Lima Scortegagna, Gabriella Zanin Fighera, Isadora Viegas, Laíse Pauletti Barp, Mateus Alonso, Alberto Vergara, Ana Maria Martins, Anete Sevciovic Grumach, Angelina Xavier Acosta, Bethania de Freitas Rodrigues Ribeiro, Camila Ferreira Ramos, Carlos Henrique Paiva Grangeiro, Chong Ae Kim, Debora Gusmão Melo, Débora Michelatto, Denise Christofolini, Ellaine Doris Fernandes Carvalho, Erlane Marques Ribeiro, Faradiba Sarquis Serpa, Flavia Resedá Brandão, Isabella Lopes Monlleó, Joyceane Alves Oliveira, Juan Clinton Llerena, Karina Carvalho Donis, Liane de Rosso Giuliani, Louise Lapagesse de Camargo Pinto, Luiz Carlos Santana da Silva, Marcela Câmara Machado Costa, Marcial Francis Galera, Marcia Maria Costa Giacon Giusti, Mara Lucia Schmitz Ferreira Santos, Maria Denise Fernandes Carvalho de Andrade, Maria Teresinha De Oliveira Cardoso, Milena Coelho Fernandes Caldato, Natalya Gonçalves Pereira, Ney Boa Sorte, Paula Frassinetti Vasconcelos de Medeiros, Paulo Ricardo Gazzola Zen, Raquel Tavares Boy da Silva, Rayana Elias Maia, Sandra Obikawa Kyosen, Solange Valle, Tatiana Amorim, Thaís Bomfim Teixeira, Vania Mesquita Gadelha Prazeres, Victor Evangelista de Faria Ferraz, Ida Vanessa Doederlein Schwartz, Domingos Alves, Têmis Maria Félix, Raras Network Group

**Affiliations:** 1 Universidade Federal de Ciências da Saúde de Porto Alegre, Porto Alegre, Brazil; 2 Programa de Pós Graduação em Saúde da Criança e do Adolescente, Universidade Federal do Rio Grande do Sul, Porto Alegre, Brasil; 3 Rede Nacional de Doenças Raras (RARAS), Porto Alegre, Brazil; 4 Medical Genetics Service, Hospital de Clínicas de Porto Alegre, Rua Ramiro Barcelos, 2350, Porto Alegre, RS 90610-910 Brazil; 5 Hospital das Clínicas da Faculdade de Medicina de Ribeirão Preto, Ribeirão Preto, Brazil; 6 Hospital Infantil João Paulo II, Belo Horizonte, Brazil; 7 Universidade Federal de São Paulo, Hospital São Paulo, São Paulo, Brazil; 8 Faculdade de Medicina do ABC, Centro Universitário FMABC, Santo André, Brazil; 9 Universidade Federal da Bahia, Hospital Universitário Prof. Edgard Santos, Salvador, Brazil; 10 Maternidade Barbara Heliodora, Rio Branco, Brazil; 11 Fundação Hospital do Acre, FUNDHACRE, Rio Branco, Brazil; 12 Hospital Universitário Walter Cantídio/UFC/EBSERH, Fortaleza, Brazil; 13 Instituto da Criança - FMUSP, São Paulo, Brazil; 14 Hospital Universitário Prof Alberto Antunes, Universidade Federal de Alagoas, Maceió, Brazil; 15 Hospital Universitário do Ceará, Universidade Estadual do Ceará, Fortaleza, Brazil; 16 Hospital Infantil Albert Sabin, Fortaleza, Brazil; 17 Hospital Santa Casa de Misericórdia de Vitória, Vitória, Brazil; 18 Centro de Referência Estadual para Assistência ao Diabetes e Endocrinologia, CEDEBA, Salvador, Brazil; 19 Instituto Nacional de Saúde da Mulher, da Criança e do Adolescente Fernandes Figueira, IFF/Fiocruz, Rio de Janeiro, Brazil; 20 Hospital Universitário Lauro Wanderley/UFPB/EBSERH, João Pessoa, Brazil; 21 Hospital Universitário Maria Pedrossian - HUMAP/UFMS/EBSERH, Campo Grande, Brazil; 22 APAE Campo Grande, Campo Grande, Brazil; 23 Hospital Infantil Joana de Gusmão, Florianópolis, Brazil; 24 Hospital Universitário Bettina Ferro de Souza, Universidade Federal do Pará, Belém, Brasil; 25 Escola Bahiana de Medicina e Saúde Pública, Salvador, Brazil; 26 Hospital Universitário Júlio Muller, Cuiabá, Brazil; 27 Instituto Jô Clemente, São Paulo, Brazil; 28 Hospital Pequeno Príncipe, Curitiba, Brazil; 29 Hospital de Apoio de Brasília, Brasília, Brazil; 30 Centro Universitário do Estado do Pará, CESUPA, Belém, Brazil; 31 Hospital Universitário da Universidade Federal de Juiz de Fora, Juiz de Fora, Brazil; 32 Unidade Acadêmica de Medicina/CCBS, Hospital Universitário Alcides Carneiro, Universidade Federal de Campina Grande, Paraíba, Brazil; 33 Hospital da Criança Santo Antônio, Universidade Federal de Ciências da Saúde de Porto Alegre, Porto Alegre, Brazil; 34 Hospital Universitário Pedro Ernesto, Universidade do Estado do Rio de Janeiro, Rio de Janeiro, Brazil; 35 Hospital Universitário Clementino Fraga Filho da UFRJ, Rio de Janeiro, Brazil; 36 APAE Salvador, Salvador, Brazil; 37 APAE Anápolis, Anápolis, Goiás Brazil; 38 Codajas-SUSAM, Manaus, Brazil; 39 Instituto Nacional de Doenças Raras, INRaras, Porto Alegre, Brazil; 40 Departamento de Genética, Universidade Federal do Rio Grande do Sul, Porto Alegre, Brazil

**Keywords:** Rare diseases, Diagnostic odyssey, Brazil, Brazilian rare diseases network, Public health

## Abstract

**Background:**

The diagnostic odyssey of individuals with rare diseases is prolonged and associated with clinical, emotional, and financial burden. In Brazil, data on diagnostic delays and their determinants remain scarce.

**Objective:**

This study aims to characterize the diagnostic odyssey of individuals with rare diseases using data from the Brazilian Rare Diseases Network (RARAS).

**Methods:**

This descriptive cross-sectional study analyzed ambispective data collected from 2018-2025 across RARAS centers nationwide. Diagnostic odyssey was defined as the time between symptom onset and definitive diagnosis. Prenatal and newborn screening diagnoses were excluded. Sociodemographic, clinical and etiological data were collected through a standardized REDCap-based instrument.

**Results:**

Among 18,625 unique participants, 12,048 had confirmed diagnoses and 5,984 met criteria for diagnostic odyssey analysis. The mean diagnostic interval was 6.21 years (± 8.41; median 2.94, IQR 0.80–8.13), indicating substantial heterogeneity. Longer delays were observed in individuals with symptom onset during adolescence. Patients referred during hospital admission experienced shorter diagnostic intervals. Regional differences were significant (*p *<0.001), with longer intervals in the Southeast region. Mean time to diagnosis ranged from 2.01 (± 2.89) years (achondroplasia) to 16.40 (± 12.59) years (hereditary angioedema). Patients consulted a mean of 5.32 (± 9.63) physicians and accessed 3.15 (± 4.96) healthcare services before diagnosis. Diagnostic intervals varied by race/ethnicity and etiological category, with longer delays among those with molecular diagnoses, while no association was observed with socioeconomic class.

**Conclusion:**

The diagnostic odyssey for rare diseases in Brazil remains prolonged and heterogeneous, reflecting structural disparities, healthcare fragmentation, and diagnostic complexity. These findings suggest that delays are not driven solely by limited access to advanced diagnostics, but also by barriers in early recognition, referral pathways, and care coordination, underscoring the need for integrated strategies across the health system.

**Supplementary Information:**

The online version contains supplementary material available at https://doi.org/10.1007/s12687-026-00939-y.

## Introduction

A diagnostic odyssey refers to the often prolonged, complex, and challenging journey that patients, especially those with rare, undiagnosed, or atypical diseases, undergo in pursuit of a definitive diagnosis. This process is typically characterized by repeated consultations with multiple healthcare providers, numerous diagnostic tests, frequent misdiagnoses, and significant delays before the correct diagnosis is established. The diagnostic odyssey can result in substantial physical, emotional, and financial burdens for patients and their families, including unnecessary interventions, hospitalizations, and psychological distress (Bordini et al. 2024; Bauskis et al. 2022; Thompson et al. 2023; Michaels-Igbokwe et al. 2021; Glaubitz et al. 2025; Domaradzki et al. 2025; Richards et al. 2015; Maksymowicz and Siwek 2024).

The term is most commonly used in the context of rare genetic disorders, where the lack of disease awareness, limited access to advanced diagnostic modalities (such as exome or genome sequencing), and fragmented care pathways contribute to the length and complexity of the journey (Bordini et al. 2024; Michaels-Igbokwe et al. 2021; Glaubitz et al. 2025; Domaradzki et al. 2025).

Worldwide, the average diagnostic delay reported for rare diseases ranges from approximately 4 to 7 years, with substantial regional and socioeconomic variation. In Europe, large patient surveys report a mean total diagnosis time of 4.7 years, with over half of patients experiencing delays greater than 6 months after first medical contact; longer delays are observed in Northern and Western regions and are influenced by factors such as age at symptom onset, sex, and the number of healthcare professionals consulted (Faye et al. 2024). Country-specific estimates within Europe are broadly consistent, including mean delays of 6.2 years in Spain (median 2 years), approximately 6 years in Germany, and 5.6 years in the United Kingdom (Benito-Lozano et al. 2022; Blöß et al. 2017; Wise et al. 2019). Outside Europe, reported delays include 7.6 years in the United States, while in China the mean delay is 4.3 years, with marked geographic and socioeconomic disparities affecting individuals in less developed regions (Wise et al. 2019; Yan et al. 2020).

The main barriers to timely diagnosis of rare diseases, particularly in low- and middle-income countries, include limited awareness and training among healthcare professionals, scarcity of diagnostic resources and infrastructure, lack of access to advanced genetic testing and insufficient numbers of specialists. Additional barriers include regulatory and logistical challenges such as difficulties in sending biological samples abroad, limited newborn screening programs, and inequities in access to diagnostic services both within and between countries. Financial constraints, lack of national strategies or policies for rare diseases, and inadequate data sharing and international collaboration further exacerbate delays. Patient advocacy groups are often underutilized, and there is a need for greater engagement and resource sharing to address these gaps (Taruscio et al. 2023; Adachi et al. 2023; Baldovino et al. 2025; Giugliani et al. 2022; Vashishta et al. 2023; Gunes et al. 2025; Fasseeh et al. 2025).

Brazil has a universal public healthcare system (Sistema Único de Saúde, SUS), organized into primary, secondary, and tertiary levels of care. Within SUS, rare disease care is structured through the National Policy for Comprehensive Care for People with Rare Diseases (PNAIPDR), established in 2014. Patients with suspected rare diseases are usually referred from primary care to accredited multidisciplinary reference centers, where specialized diagnostic investigation and follow-up are provided. However, patients may also enter the diagnostic pathway through secondary care or hospital admission, particularly when presenting with severe or acute manifestations. The Brazilian Unified Health System (SUS) funds the majority of diagnostic and treatment services for rare diseases. Although the implementation of this policy has expanded access to specialized care, coverage and integration remain incomplete, particularly outside major urban centers. Genetic services, laboratory infrastructure, and medical genetics expertise remain unevenly distributed across the country, with greater concentration in the South and Southeast regions. Consequently, many patients require referral to distant centers, contributing to delays in diagnosis and follow-up, while challenges in care coordination persist across different levels of the health system (Horovitz et al. 2024; Félix et al. 2023a, b; de Oliveira et al. 2024; Brasil 2026).

Despite these advances, the magnitude and determinants of the diagnostic odyssey remain poorly characterized at the national level. Previous analyses from the Brazilian Rare Diseases Network (RARAS) reported a mean diagnostic delay of 5.4 years between symptom onset and diagnosis, with important variation according to disease type, geographic region, and access to specialized services. These findings reflect persistent barriers, including limited awareness among healthcare professionals, unequal access to molecular diagnostic technologies, and regional disparities in specialized care (de Oliveira et al. 2024; Lopes et al. 2018).

For specific rare diseases, such as Friedreich’s ataxia, the mean diagnostic delay can be even longer in Brazil, reaching 7.8 years and involving visits to an average of 6.2 physicians prior to diagnosis (Machado et al. 2024). Notably, a previous study in Brazil reported that the diagnostic odyssey for mucopolysaccharidosis lasted 4.8 years (Vieira et al. 2008). To address this gap, the present study aimed to characterize the diagnostic odyssey and investigate factors associated with diagnostic delay in rare diseases in Brazil using data from the Brazilian Rare Diseases Network (RARAS). Although previous analyses within the RARAS network have addressed aspects of the diagnostic pathway, this is the first nationwide study specifically designed to comprehensively characterize the diagnostic odyssey in Brazil.

## Methods

This descriptive cross-sectional study analyzed data from the Brazilian Rare Diseases Network (RARAS), a multicenter epidemiological initiative aimed at characterizing rare diseases across Brazil (Félix et al. 2022). An ambispective survey design was adopted. In the retrospective phase, data were collected from individuals under diagnostic investigation or with a confirmed or suspected rare disease who were evaluated between 2018 and 2019 at thirty-four participating centers. In the prospective phase, data collection was conducted from 2022 to April 2025, during which the network expanded to include 38 participating centers nationwide. Data were obtained through review of medical records in both phases and complemented by direct interviews with participants during the prospective phase. The standardized data collection instrument is publicly available through the LattesData platform (Félix et al. 2023a, b). The form captured demographic, clinical, and therapeutic information and was implemented using the Research Electronic Data Capture (REDCap) platform, hosted at Ribeirão Preto Medical School, University of São Paulo as described by Alves et al. (2021).

Demographic variables included age, sex, race (according to the official classification of the Brazilian Institute of Geography and Statistics, IBGE), and region of residence. Among participants with a calculated diagnostic odyssey and available socioeconomic classification (*n* = 3,848), classes A and B1 were grouped due to the small number of individuals in class A (A = 10; B1 = 81), and classes D and E were combined given their similar socioeconomic profile.

To ensure data quality and standardization across centers with heterogeneous professional backgrounds, training sessions were provided to all participating teams prior to data collection.

Phenotypic features were encoded using the Human Phenotype Ontology (HPO), with a maximum of five terms recorded per individual. Diagnostic information was documented using ORPHA codes.

For the purposes of this study, the diagnostic odyssey was defined as the elapsed time between the onset of the first reported symptom and the establishment of a definitive diagnosis. Age at symptom onset was converted into a calendar date and subtracted from the recorded date of diagnosis to calculate the diagnostic odyssey, expressed in years.

**Fig. 1:**
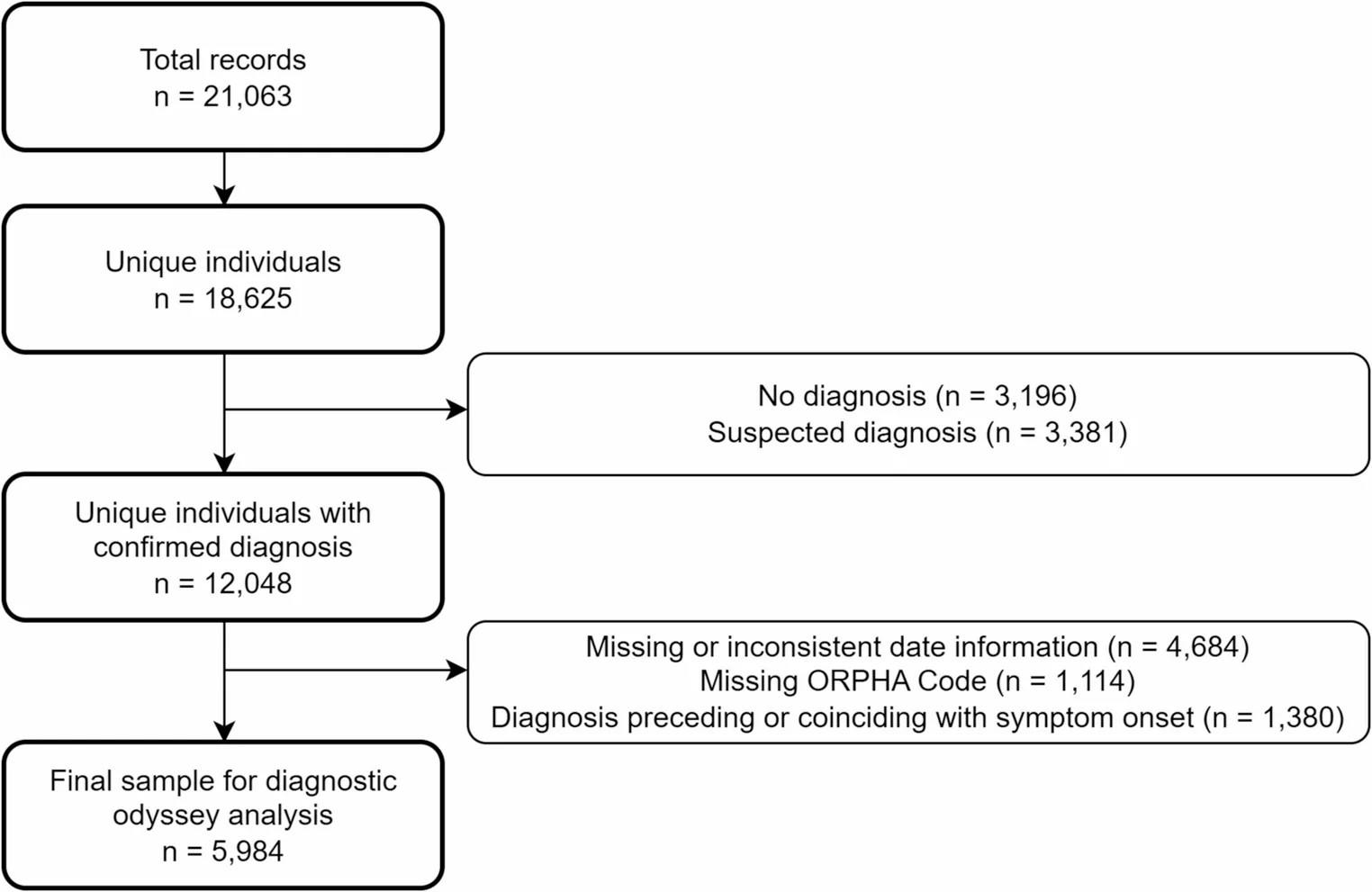
Study population selection flowchart

The consolidated database comprised 21,063 records corresponding to 18,625 unique individuals, of whom 12,048 had a confirmed diagnosis. After applying eligibility criteria, 5,984 individuals were included in the diagnostic odyssey analysis. The full process of inclusion and exclusion is detailed in Fig. 1. Cases in which diagnosis preceded or coincided with symptom onset, including those identified through prenatal or newborn screening, were excluded, as these do not constitute a diagnostic odyssey.

Statistical analyses were performed using R software (version 4.4.1). Continuous variables are presented as means and medians with corresponding standard deviations and interquartile range. Categorical variables are presented as counts and percentages. Descriptive analyses were stratified according to age group, region of residence, and the most frequently reported rare diseases in the dataset. Quantitative measures were compared between groups using the Kruskal-Wallis test. Post hoc pairwise comparisons were conducted using Dunn’s test with Bonferroni correction, with statistical significance defined as *p* < 0.05/2. To estimate the expected age at diagnosis at the population level, including individuals who remained undiagnosed, Kaplan–Meier analysis was performed, considering birth as time zero and undiagnosed individuals as right-censored.

## Results

Among individuals included in the diagnostic odyssey analysis (*n* = 5,984), 53.23% were female, 46.71% male, and 0.06% undefined. The mean age at data collection was 22.35 years (± 19.12; median 15.74; IQR 7.30–33.95; range: 0.01–93.93). Most participants were born in the Northeast region (41.86%), followed by the Southeast (25.77%); a similar distribution was observed for the region of residence (Northeast 42.46%; Southeast 26.50%) (Table 1).

**Table 1:** Population, number of patients, and patients per million inhabitants represented in RARAS by Brazilian region. Population estimates were obtained from the Brazilian Institute of Geography and Statistics (IBGE)

Region	Population (IBGE)	Patients	Patients per million inhabitants
Northeast	57,112,096	2504	43.84
South	31,113,021	1160	37.28
Midwest	17,071,595	365	21.38
Southeast	88,617,693	1544	17.42
North	18,801,282	235	12.50

The five most prevalent phenotypic features in this sample were global developmental delay (HP:0001263) (*n* = 484), followed by short stature (HP:0004322) (*n* = 385), seizure (HP:0001250) (*n* = 339), hypotonia (HP:0001252) (*n* = 288), and muscle weakness (HP:0001324) (*n* = 241).

**Fig. 2:**
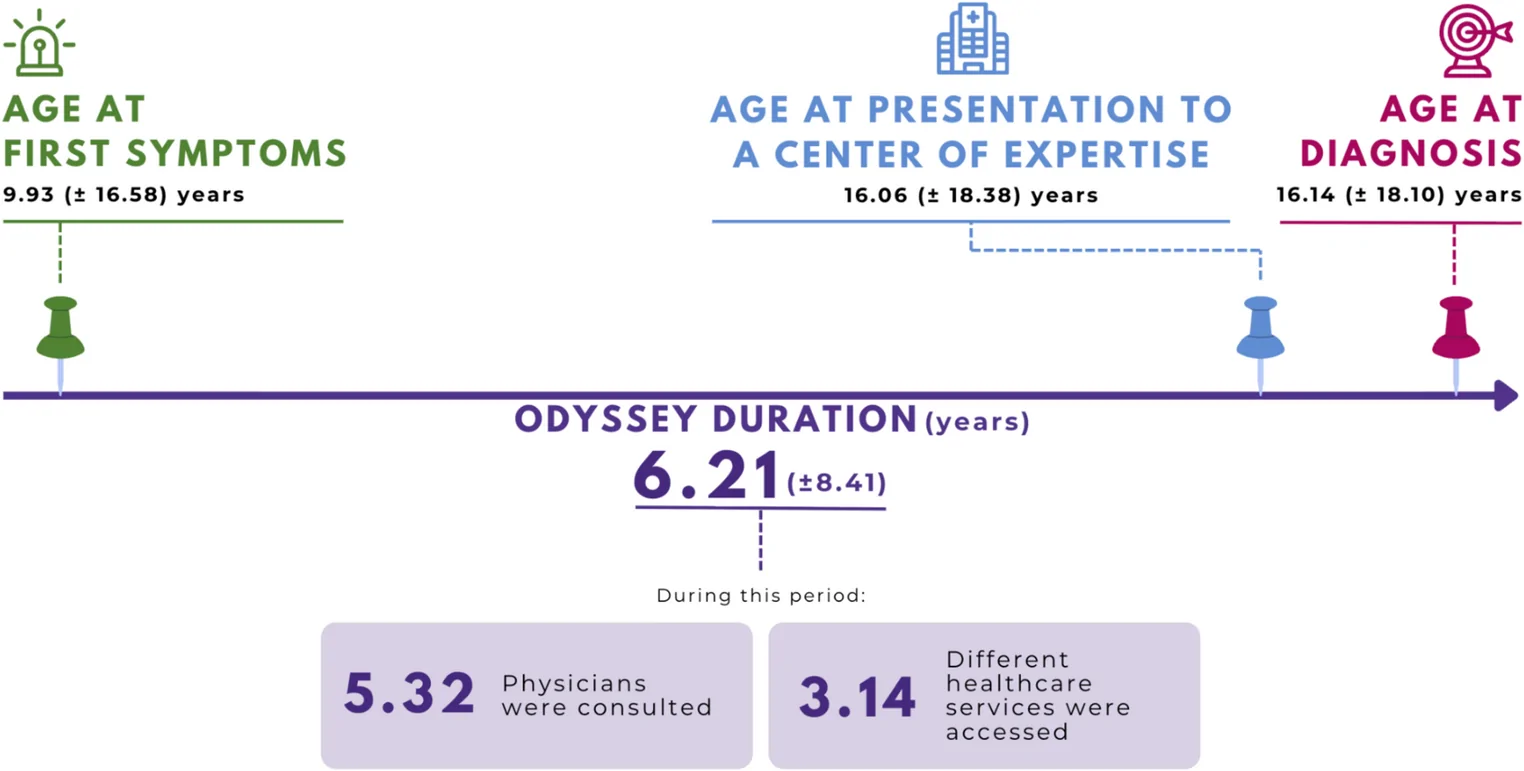
Mean (± SD) diagnostic odyssey (years), mean number of physicians consulted and healthcare services accessed before diagnosis

**Fig. 3:**
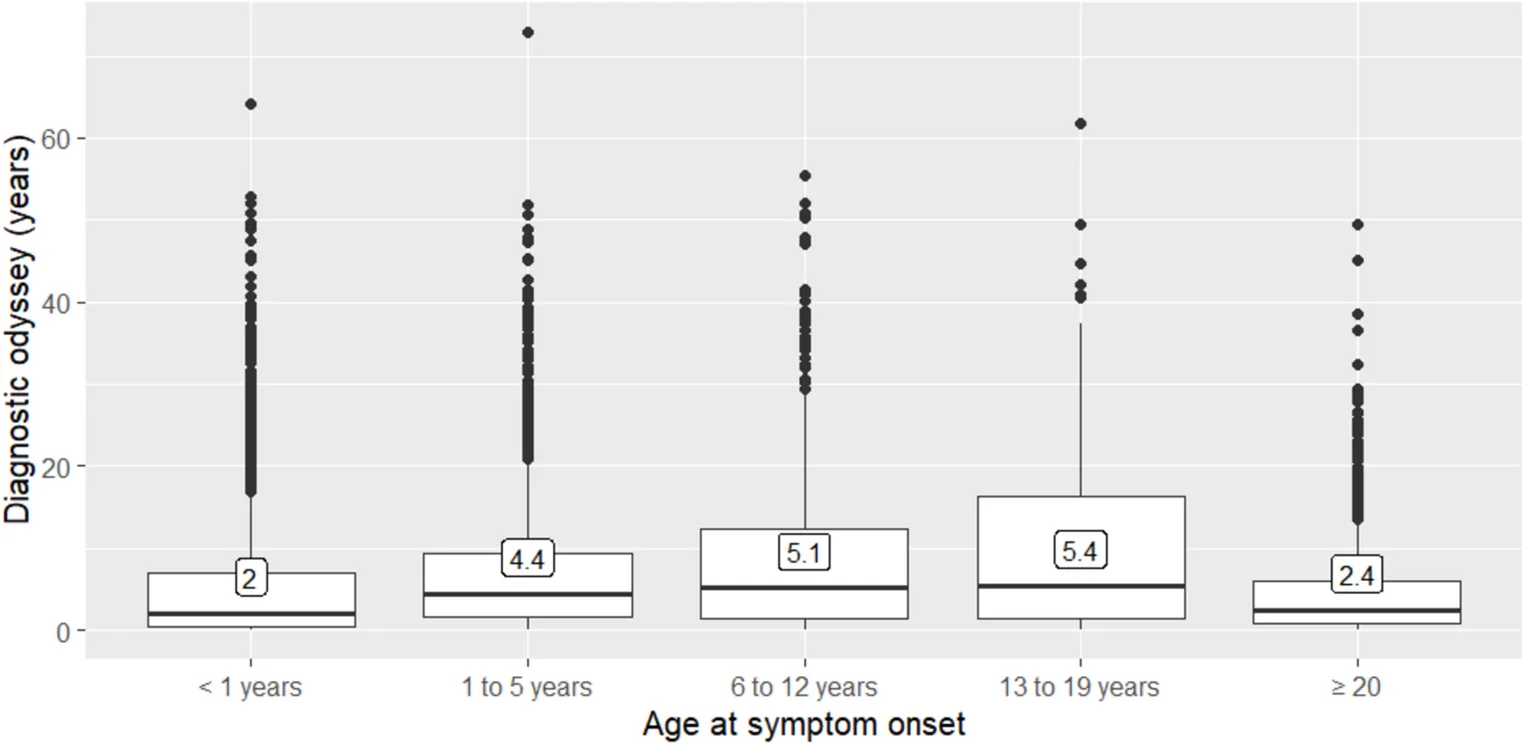
Median diagnostic odyssey (in years) by age at symptom onset

The overall mean time between symptom onset and diagnosis was 6.21 years (± 8.41; median: 2.94; IQR: 0.80–8.13) (Fig. 2). The diagnostic interval showed marked variability, as reflected by the high standard deviation, indicating substantial heterogeneity in individual diagnostic trajectories. Participants whose symptom onset occurred between 13 and 19 years of age experienced longer diagnostic odysseys. Figure 3 summarizes the median diagnostic odyssey according to the age at onset of signs and symptoms (Suppl. 1). A total of 78 individuals had symptom onset at 65 years of age or older.

**Fig. 4:**
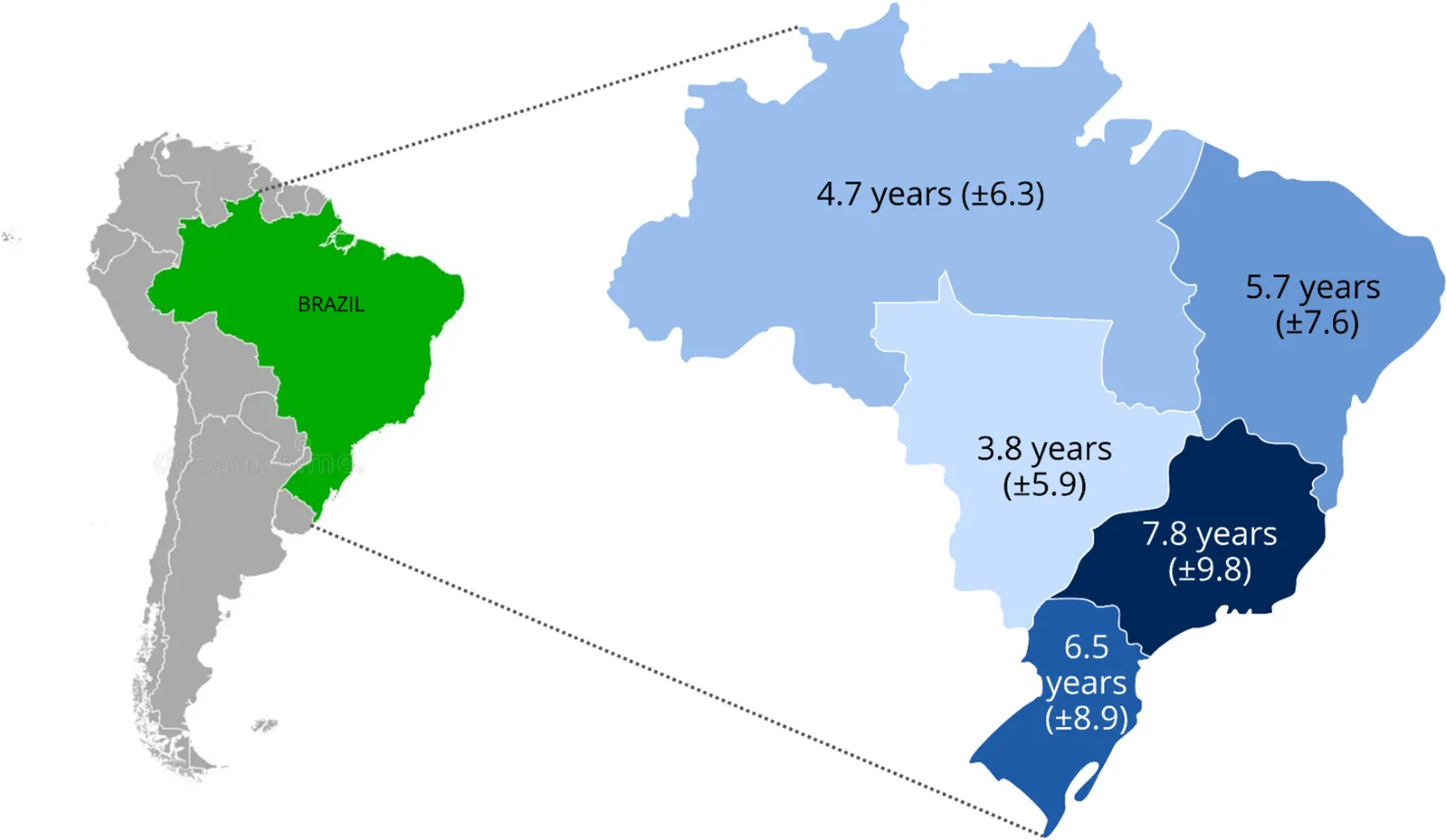
Average (± SD) diagnostic odyssey stratified by region of birth. Sample sizes by region were as follows: North (*n* = 235), Northeast (*n* = 2,504), Midwest (*n* = 365), Southeast (*n* = 1,544), and South (*n* = 1,160)

Regional differences in average time to diagnosis were observed when stratified by region of birth (Fig. 4). The diagnostic odyssey differed significantly according to region of birth (Kruskal–Wallis test, *p* < 0.001), excluding cases with missing regional information, indicating heterogeneity in diagnostic intervals across regions. *Post hoc* pairwise comparisons using Dunn’s test with Bonferroni correction identified significant differences between specific regions. The Southeast region showed significantly longer diagnostic odysseys compared with all other regions (Center-West, Northeast, North, and South) (*p* < 0.001).

Among the most frequent diseases analyzed, the longest odysseys were observed in hereditary angioedema (16.40 ± 12.59 years), Fabry disease (12.13 ± 11.56 years) and osteogenesis imperfecta type 1 (11.73 ± 13.90 years). The shortest time to diagnosis was seen in achondroplasia (2.01 ± 2.89 years) (Table 2). A wide range of diagnostic delays was observed across conditions, with mean times to diagnosis ranging from just over one year to more than sixteen years.

**Table 2:** Mean and median diagnostic odyssey for the most frequent rare diseases

Rare disease	Mean (years)	SD	Median	IQR	*N*
Hereditary angioedema	16.40	12.59	13.50	5.98–24.61	132
Fabry disease	12.13	11.56	9.04	2.23–21.12	43
Osteogenesis imperfecta, type 1	11.73	13.90	6.79	1.80–18.57	83
Marfan syndrome	10.28	10.97	6.54	2.75–14.34	63
Neurofibromatosis type 1	9.75	10.90	5.80	2.00–14.12	217
Gaucher disease type 1	9.25	10.42	5.10	1.38–13.50	46
Noonan syndrome	6.78	7.98	3.23	1.27–10.39	58
Familial amyloid polyneuropathy	5.94	8.45	2.97	1.23–5.61	45
Turner syndrome	5.63	7.57	2.82	0.74–7.26	183
Acromegaly	5.39	7.20	2.81	1.31–5.79	70
Mucopolysaccharidosis type 2	5.03	7.00	3.01	1.08–5.32	70
Muscular dystrophy, Duchenne type	4.93	4.27	3.99	1.74–6.86	175
Cystic fibrosis	4.33	8.00	0.69	0.14–4.78	54
Myasthenia gravis	4.24	6.73	1.74	0.66–3.87	56
Mucopolysaccharidosis type 6	3.27	5.44	1.48	0.75–3.49	49
Amyotrophic lateral sclerosis	2.96	3.38	2.10	1.20–3.40	100
Systemic lupus erythematosus	2.95	5.30	1.00	0.55–2.34	126
Achondroplasia	2.01	2.89	0.79	0.25–2.97	79

*SD* standard deviation, *N* sample size.

The comparison of mean diagnostic odysseys by region, before and after excluding individuals with hereditary angioedema, showed overall stability, although the exclusion had a greater impact in the Southeast. No changes were observed in the Midwest, North, and South regions, while a slight reduction was noted in the Northeast (5.67 to 5.62 years). In contrast, the Southeast showed the largest decrease (7.80 to 7.09 years), indicating a relevant contribution of hereditary angioedema to longer delays in this region; however, it remained the region with the longest diagnostic odyssey even after exclusion.

As indicators of healthcare fragmentation during the diagnostic trajectory, individuals consulted an average of 5.32 (± 9.63) physicians and accessed 3.15 (± 4.96) different healthcare services (Fig. 2). Notably, 14.5% of patients visited five or more healthcare services, while 16.68% consulted five or more physicians before receiving a definitive diagnosis.

The duration of the diagnostic odyssey differed significantly according to the referral source (Kruskal–Wallis, *p* < 0.001). Patients referred during hospital admission had the shortest diagnostic odyssey (mean 2.8 ± 5.1 years), followed by those referred through emergency departments (4.6 ± 7.1 years). Longer diagnostic intervals were observed among patients referred from outpatient clinics (6.6 ± 8.5 years) and primary care (6.8 ± 8.9 years), while referrals classified as other sources presented the longest delay (9.0 ± 11.3 years).

Individuals residing outside the city where the specialized center was located had a longer diagnostic interval compared to those living in the same city (mean 6.47 ± 8.58 vs. 5.73 ± 8.06 years; median 3.19 vs. 2.54 years). This difference was statistically significant (Kruskal–Wallis test, *p* < 0.001).

Among participants with available socioeconomic classification according to the Brazilian Association of Research Companies (ABEP) criteria (*n* = 3,848), no significant differences in diagnostic odyssey duration were observed across socioeconomic classes, with mean times ranging from 6.24 to 8.32 years (Suppl. 2).

Race/ethnicity data were available for 5,735 of the 5,984 participants with a calculated diagnostic odyssey. Diagnostic odyssey duration differed significantly across race/ethnicity categories (Kruskal–Wallis test, *p* < 0.001), excluding Indigenous participants due to small sample size (*n* = 20). Dunn’s post hoc analysis with Bonferroni adjustment showed that White individuals had longer diagnostic odysseys than Brown individuals (*p* < 0.001) and shorter odysseys than Black individuals (*p* = 0.022). Black individuals also experienced longer diagnostic odysseys than Brown individuals (*p* < 0.001). No other pairwise differences were significant (Suppl. 2).

Diagnostic odyssey duration differed significantly across diagnostic categories (Kruskal–Wallis test, *p* < 0.001). Mean odyssey duration was longest among patients with molecular etiological diagnoses (7.18 ± 8.26 years), followed by cytogenetic diagnoses (5.88 ± 7.34 years), and shortest among those with biochemical diagnoses (4.03 ± 7.08 years). Patients with clinical diagnoses showed intermediate durations (Suppl. 2). *Post hoc* pairwise comparisons using Dunn’s test with Bonferroni correction confirmed that clinical diagnoses were associated with longer odysseys than biochemical diagnoses and shorter odysseys than molecular diagnoses. Biochemical diagnoses were associated with shorter odysseys than both cytogenetic and molecular diagnoses, and cytogenetic diagnoses showed shorter odysseys than molecular diagnoses. All reported differences met the predefined threshold for statistical significance.

Kaplan–Meier analysis of age at diagnosis, including undiagnosed individuals as right-censored, estimated a projected median age at diagnosis of 23.57 years. This analysis included 8,476 individuals with a confirmed diagnosis and 6,575 without a diagnosis, thus capturing a broader population than the diagnostic odyssey analysis. In contrast, the observed median age at diagnosis among diagnosed individuals was 8.72 years. This difference reflects the impact of censoring and the inclusion of undiagnosed individuals, which shifts the population-level estimate toward older ages. When multiple diagnostic dates were available, the earliest was considered. Numbers at risk and incident diagnoses are presented in Supplementary Fig. 3.

### Extreme diagnostic delays

To characterize extreme diagnostic delays, we examined individuals above the 99th percentile of diagnostic odyssey duration, corresponding to delays ≥ 38.78 years (*n* = 60). This threshold was selected to capture the upper tail of the distribution while preserving clinical interpretability. Within this subgroup, hereditary angioedema (*n* = 8), neurofibromatosis type 1 (*n* = 5), osteogenesis imperfecta (*n* = 7), Turner syndrome (*n* = 3), and Marfan syndrome (*n* = 2) were the most frequently represented conditions; all other diseases occurred only once.

In this extreme-delay group, the mean age at symptom onset was 5.39 ± 6.65 years, whereas the mean age at diagnosis was 51.59 ± 9.46 years, highlighting prolonged diagnostic trajectories extending across decades. Most patients resided in the Southeast (*n* = 25), followed by the North (*n* = 18) and South (*n* = 12) regions.

#### Discussion and conclusions

The study’s average time to diagnosis was 6.21 years, aligning with European and North American cohorts reporting averages between 4.7 and 7.6 years of odyssey (Faye et al. 2024; Benito-Lozano et al. 2022; Wise et al. 2019). The high standard deviation reflects substantial heterogeneity in clinical presentation and disease trajectories across rare conditions, limiting direct comparisons (Thompson et al. 2023). Table 3 provides an integrated overview of the systemic determinants, key findings, and healthcare implications discussed throughout this section.

**Table 3:** Summary of systemic determinants, key findings and healthcare implications of the diagnostic odyssey for rare diseases in Brazil

Finding	Possible explanation	Implications
Longer odyssey in adolescents	Less specific manifestations; transition between pediatric and adult care	Improve recognition of late-onset rare diseases and transition programs
Regional differences	Unequal distribution of specialized services and referral centers	Expand specialized care and improve regional referral networks
Longer odyssey among individuals living outside the referral city	Geographic barriers and travel to specialized centers	Strengthen regional networks and decentralize expertise
Shorter odyssey among hospitalized patients	Greater disease severity and earlier access to specialists	Earlier referral pathways may reduce delays
Longer odyssey for referrals from primary care/outpatient clinics	Multiple referrals before reaching expertise	Improve rare disease training in primary care
Longer odyssey for molecular diagnoses	Limited access to genomic testing during much of the study period	Expand equitable access to genomic diagnostics
Racial disparities	Structural inequities affecting access to specialized care	Policies promoting equitable access
High variability among diseases	Different phenotypes, disease severity and diagnostic complexity	Disease-specific diagnostic strategies
Regional differences in RARAS patient representation	Higher representation in Northeast (43.84 pts/million) vs. North (12.50 pts/million)	May reflect differences in access to specialized care, regional referral patterns, and variation in patient capture by the RARAS network
Uneven medical specialist distribution	Extreme concentration of geneticists in Southeast (148) vs. North (7)	May contribute to geographic barriers and regional referral bottlenecks
No significant association with socioeconomic status	The sample is restricted to individuals who already successfully accessed specialized SUS centers, which may result in more homogeneous trajectories among those who overcome initial entry barriers	May reflect an equalizing effect of specialized SUS care among those who reach referral centers, while not capturing socioeconomic barriers before referral or survival bias among those who do not reach specialized care.

A crucial finding was the longer diagnostic odyssey among individuals with adolescent symptom onset, consistent with European data (Faye et al. 2024). This may reflect the lower specificity of early manifestations in late-onset rare diseases and the transition from pediatric to adult healthcare, which is often associated with fragmented care and limited structured support (Machado et al. 2024; Sandquist et al. 2022). These findings reinforce the need to improve awareness of late-onset rare diseases and train healthcare professionals to recognize warning signs beyond early childhood (Gunes et al. 2025; Bauskis et al. 2022).

Observed regional, socioeconomic, and racial disparities reinforce that the diagnostic odyssey is a reflection of systemic inequities in healthcare access rather than a purely clinical phenomenon. The Southeast region, despite concentrating a large share of specialized services, human and diagnostic resources, showed longer diagnostic intervals (Horovitz et al. 2013, 2024; de Oliveira et al. 2023). This likely reflects the greater complexity of care pathways in resource-rich settings, where patients often undergo multiple referrals before reaching specialized centers. The region may also function as a referral hub, concentrating more complex and atypical cases, which may be associated with longer diagnostic trajectories. In addition, fragmentation of care, poorly coordinated referral pathways and limited awareness of rare diseases among non-specialist clinicians may contribute to delays, even where resources are available (Faye et al. 2024; Willmen et al. 2023). From an epidemiological perspective, demographic and structural differences in birth rates, internal migration, and population density may strongly influence case detection and patients’ access to the healthcare system. Although the Northeast accounted for the highest proportion of births (41.86%) and current residence (42.46%) in this cohort, interregional migration to specialized services may concentrate the most complex and atypical cases in the major urban centers of the Southeast (Castro et al. 2021). These results indicate that the availability of resources alone does not ensure timely diagnosis and highlight the importance of care integration and efficient referral systems. The absence of regional differences in the North should be interpreted with caution, as access barriers and possible underrepresentation of indigenous, *quilombola*, and riverine populations may not be fully captured in this sample. Future analyses within the RARAS network should explore patient mobility to better understand whether complex cases are being concentrated in specific regions.

Patients referred during hospital admission had the shortest diagnostic odyssey, likely reflecting greater disease severity and complexity, which may trigger multidisciplinary assessment and faster access to specialists and diagnostic resources. This finding should not be interpreted as a protective effect of hospitalization itself, but rather as a reflection of the clinical characteristics of patients requiring inpatient care (Thompson et al. 2023; Willmen et al. 2023; Faye et al. 2024).

In line with these findings, geographic proximity to specialized centers also influenced diagnostic timing at the individual level. Individuals residing outside the city where the specialized center was located experienced longer diagnostic intervals, reinforcing geographic access as a key determinant of the diagnostic odyssey. This aligns with evidence showing that distance from specialized services and the need to travel for expert evaluation are associated with increased delays. In Spain, patients diagnosed outside their region or requiring interprovincial travel had more than twice the odds of delay (Benito-Lozano et al. 2022), while studies in Italy and Germany demonstrated that place of residence and distance to tertiary centers independently influence diagnostic timing, described as the “postcode lottery effect” (Cirillo et al. 2025; Walter et al. 2021).

These findings are consistent with the current distribution of specialized genetic services and the medical genetics workforce in Brazil. Although the number of medical geneticists has increased by more than 100% over the past decade, specialized services and referral centers remain concentrated in large urban areas, particularly in the South and Southeast regions (Scheffer 2025). According to the latest Brazilian Medical Workforce data, 376 medical geneticists are currently registered nationwide, with 55.7% concentrated in the Southeast and South (148 and 83, respectively) and only 7 in the North (Scheffer 2025). Moreover, most medical geneticists practice in state capitals, potentially limiting access for patients living in smaller municipalities and underserved areas (Horovitz et al. 2024; Félix et al. 2022; de Oliveira et al. 2024). This persistent geographic concentration of specialized professionals may contribute to the longer diagnostic pathways observed among patients outside major referral centers.

The reported diagnostic interval may underestimate the national reality, as the study population is drawn from specialized centers and therefore represents patients who accessed referral care (de Oliveira et al. 2024). This sample likely overrepresents individuals who successfully reached specialized services, potentially underestimating diagnostic delays among patients who remain undiagnosed in primary care settings or who are never referred to a specialized center. Survival bias may also contribute, as individuals with severe or rapidly progressive diseases may die before reaching specialized care or receiving a definitive diagnosis, particularly in settings with greater barriers to access. Consequently, the true diagnostic odyssey in the broader Brazilian population may be even longer.

Diagnostic delays are particularly pronounced in conditions characterized by nonspecific or atypical manifestations, variable expressivity, intermittent symptoms, or overlapping phenotypes, which hinder clinical recognition and often require specialized testing. This is consistent with our findings, as the most frequently reported HPO terms in the cohort were predominantly nonspecific, potentially contributing to delayed recognition. Conversely, disorders presenting with distinctive or visible phenotypic features, such as achondroplasia, or those identifiable through biochemical screening, tend to achieve earlier diagnosis (Maksymowicz and Siwek 2024; Kuiper et al. 2018; Benito-Lozano et al. 2022; Zurynski et al. 2017). Hereditary angioedema illustrates the consequences of these diagnostic challenges: its heterogeneous and intermittent manifestations frequently mimic common allergic or surgical conditions, and in Brazil it has been associated with a mean diagnostic delay of 12.9 years in a national cohort of more than 800 patients (Ferriani et al. 2025). Low clinical suspicion, normalization of recurrent symptoms, misinterpretation of prodromal signs, and limited access to confirmatory testing may further contribute to delayed recognition (Isono et al. 2022; Busse and Christiansen 2020).

The diagnostic odyssey in Brazil is characterized by multi-year delays, multiple specialist consultations, and significant systemic barriers, including limited access to specialized care and insufficient professional training in rare disease recognition (de Oliveira et al. 2024; Lopes et al. 2018; Machado et al. 2024; Horovitz et al. 2024). Evidence shows that generalist doctors may struggle to recognize rare diseases, contributing to misdiagnosis and fragmented care (Thompson et al. 2023; Maksymowicz and Siwek 2024). This underscores the need for continuous professional education and greater integration of rare diseases into medical curricula, with training focused on early recognition and support for families (Gunes et al. 2025; Bauskis et al. 2022). Beyond its impact on patients and families, prolonged diagnostic pathways may also result in inefficient use of healthcare resources before diagnosis (Glaubitz et al. 2025).

Internationally, lower socioeconomic status is associated with longer diagnostic delays. In our sample, however, socioeconomic status was not significantly associated with diagnostic delay. This finding should be interpreted cautiously, as all participants had accessed specialized services within the Brazilian public health system, which may have resulted in more homogeneous diagnostic pathways across socioeconomic strata. Nevertheless, broader social and structural barriers may still influence diagnostic access and care coordination (Yan et al. 2020; Taruscio et al. 2023; Horovitz et al. 2024).

Significant differences in diagnostic odyssey were observed across racial groups, with longer intervals among Black individuals, followed by White and Brown individuals. The longer delays among Black individuals are consistent with previous evidence and may reflect structural barriers, inequities in access to specialized care, and institutional racism (Sato et al. 2026; de Oliveira et al. 2024; Horovitz et al. 2024). International studies similarly show longer diagnostic delays and lower rates of diagnosis among racial and ethnic minorities, supporting a predominantly systemic rather than biological explanation (Richards et al. 2015; Wojcik et al. 2023; Borja et al. 2025). Potential mechanisms may also operate within genomic and diagnostic technologies, as individuals of non-European ancestry are more likely to harbor variants of uncertain significance, reflecting their underrepresentation in genomic databases, while facial phenotyping technologies may be affected by the underrepresentation of ancestrally diverse populations in training datasets (Dawood et al. 2024; Kruszka and Tekendo-Ngongang 2023). The pattern observed among White and Brown individuals is less commonly reported and should therefore be interpreted cautiously, as differences in regional distribution, referral dynamics, and case mix may have contributed to this finding. Thus, the observed racial differences may partly reflect regional distribution, geographic access, referral patterns, and case mix rather than an independent effect of race. Because racial composition and access to specialized services vary geographically in Brazil, regional differences may partly contribute to the observed racial patterns. Further multivariable analyses incorporating geographic and healthcare-access variables are needed to clarify these associations (Faugno et al. 2025; Tang et al. 2026).

Although expanding access to genetic diagnostic tests remains important, a substantial portion of the diagnostic delay likely occurs prior to referral to specialized centers, highlighting the critical role of primary care and early clinical recognition. Strengthening training and awareness among primary care professionals is therefore essential, as early recognition and timely referral may significantly reduce the diagnostic odyssey (Faye et al. 2024; Blöß et al. 2017).

Among patients who ultimately received a molecular etiological diagnosis, longer diagnostic odysseys were observed, likely reflecting limited access to molecular testing rather than lower diagnostic yield. Although next-generation sequencing has recently been incorporated into clinical practice in Brazil in the Public Health System, access remains uneven, with exome sequencing in the public health system still restricted to intellectual disability (de Oliveira et al. 2023). Patients therefore often undergo multiple previous investigations before genomic testing becomes available. Approximately 35% of individuals evaluated in specialized centers still lack a definitive diagnosis, as previously reported in the RARAS network, reinforcing the need to expand equitable access to comprehensive diagnostic strategies (Bordini et al. 2024; Wise et al. 2019; de Oliveira et al. 2024).

Reducing the diagnostic odyssey requires strengthening primary care capacity for early recognition and appropriate referral. The implementation of clear referral pathways can facilitate earlier diagnosis (Domaradzki et al. 2025), while expanding newborn screening may help prevent prolonged diagnostic trajectories in selected conditions (Nakar-Weinstein et al. 2025). The incorporation of genomic technologies can further support diagnosis, particularly in complex cases, but should be implemented alongside clear clinical guidelines (Wise et al. 2019; Giugliani et al. 2022; Félix et al. 2023a, b).

Although our findings are consistent with the existing literature, some limitations must be acknowledged. Eligibility criteria for the diagnostic odyssey analysis restricted the sample to approximately half of confirmed cases, mainly due to missing key dates required to calculate the diagnostic interval. Cases diagnosed prenatally or through newborn screening, as well as those in which the diagnosis occurred before or at the time of symptom onset, were excluded. These situations represent distinct clinical pathways in which the traditional concept of a diagnostic odyssey does not apply, as newborn screening programs are specifically designed to enable early detection and prevent prolonged diagnostic trajectories (Giugliani et al. 2022; Vashishta et al. 2023; Gunes et al. 2025; Fasseeh et al. 2025). In addition, incomplete data and heterogeneity in documentation are inherent challenges in retrospective analyses of rare disease registries and may introduce selection bias related to data completeness and quality (Benito-Lozano et al. 2022; Blöß et al. 2017; Wise et al. 2019; Yan et al. 2020; Taruscio et al. 2023; Adachi et al. 2023; Baldovino et al. 2025). These results may also reflect sampling characteristics or patterns of access to referral centers rather than true differences across groups. Future studies could address this limitation by applying cluster-based approaches, considering the type of center as a clustering variable. Because the study was based on referral centers, disease-specific prevalence estimates cannot be interpreted as population frequencies.

This nationwide, multicenter analysis provides real-world evidence that the diagnostic odyssey for rare diseases in Brazil remains prolonged, highly heterogeneous, and structurally influenced. To our knowledge, this is the first study to comprehensively characterize its determinants within a coordinated national network. Beyond overall delays, the findings highlight substantial fragmentation of care, with patients navigating multiple professionals and services before diagnosis, as well as significant geographic and racial disparities. Together, these results indicate that delayed diagnosis is not solely driven by technological limitations, but largely reflects systemic barriers in access, coordination, and early clinical recognition.

These findings underscore the need for coordinated public policies, strengthened referral pathways, and improved integration of care across the health system. Expanding equitable access to diagnostic technologies remains important but should be accompanied by investment in professional training and awareness to promote earlier recognition and timely referral. Ultimately, reducing the diagnostic odyssey will depend not only on expanding access to genomic technologies but also on strengthening referral pathways, and coordination of care throughout the healthcare system.

## Supplementary Information

Below is the link to the electronic supplementary material.

Supplementary Material 1 (PNG 903 KB)

Supplementary Material 2 (XLSX 145 KB)

Supplementary Material 3 (PNG 88.6 KB)

## Data Availability

Data analyzed in this study are available interactively through the Brazilian Online Atlas of RD (RARASBR, https://doi.org/10.25504/FAIRsharing.d7b6c8). For any further inquiries, please contact the corresponding author.
